# IMU Sensor-Based Worker Behavior Recognition and Construction of a Cyber–Physical System Environment

**DOI:** 10.3390/s25020442

**Published:** 2025-01-13

**Authors:** Sehwan Park, Minkyo Youm, Junkyeong Kim

**Affiliations:** Advanced Institute of Convergence Technology, 145 Gwanggyo-ro, Yeongtong-gu, Suwon-si 16229, Gyeonggi-do, Republic of Korea; sehwan0721@snu.ac.kr

**Keywords:** IMU sensor, behavior recognition, real-time monitoring, CPS

## Abstract

According to South Korea’s Ministry of Employment and Labor, approximately 25,000 construction workers suffered from various injuries between 2015 and 2019. Additionally, about 500 fatalities occur annually, and multiple studies are being conducted to prevent these accidents and quickly identify their occurrence to secure the golden time for the injured. Recently, AI-based video analysis systems for detecting safety accidents have been introduced. However, these systems are limited to areas where CCTV is installed, and in locations like construction sites, numerous blind spots exist due to the limitations of CCTV coverage. To address this issue, there is active research on the use of MEMS (micro-electromechanical systems) sensors to detect abnormal conditions in workers. In particular, methods such as using accelerometers and gyroscopes within MEMS sensors to acquire data based on workers’ angles, utilizing three-axis accelerometers and barometric pressure sensors to improve the accuracy of fall detection systems, and measuring the wearer’s gait using the x-, y-, and z-axis data from accelerometers and gyroscopes are being studied. However, most methods involve use of MEMS sensors embedded in smartphones, typically attaching the sensors to one or two specific body parts. Therefore, in this study, we developed a novel miniaturized IMU (inertial measurement unit) sensor that can be simultaneously attached to multiple body parts of construction workers (head, body, hands, and legs). The sensor integrates accelerometers, gyroscopes, and barometric pressure sensors to measure various worker movements in real time (e.g., walking, jumping, standing, and working at heights). Additionally, incorporating PPG (photoplethysmography), body temperature, and acoustic sensors, enables the comprehensive observation of both physiological signals and environmental changes. The collected sensor data are preprocessed using Kalman and extended Kalman filters, among others, and an algorithm was proposed to evaluate workers’ safety status and update health-related data in real time. Experimental results demonstrated that the proposed IMU sensor can classify work activities with over 90% accuracy even at a low sampling rate of 15 Hz. Furthermore, by integrating internal filtering, communication modules, and server connectivity within an application, we established a cyber–physical system (CPS), enabling real-time monitoring and immediate alert transmission to safety managers. Through this approach, we verified improved performance in terms of miniaturization, measurement accuracy, and server integration compared to existing commercial sensors.

## 1. Introduction

According to the Occupational Safety and Health Administration (OSHA), the construction industry accounted for 5486 fatal work injuries in 2022, equating to a rate of 3.7 fatalities per 100,000 full-time equivalent workers [1]. Similarly, the Health and Safety Executive (HSE) in Great Britain reports that the construction sector continues to account for the greatest number of workers killed in fatal accidents each year [2]. Common accident types include falls, slips, crush injuries, entanglements, and collisions. Particularly, workers at construction sites are exposed to various risks such as collisions with construction equipment, falls from heights, and slips. Most safety accidents occurring at construction sites are caused not by unsafe facilities and infrastructure but by unsafe work practices of the workers themselves.

Various studies are being conducted to prevent accidents resulting from such unsafe work practices. One of these technologies involves monitoring techniques using computer vision (CV), which is being developed by training and applying monitoring systems through advancements in deep learning technology. However, there are areas that CV technology cannot cover. Monitoring accuracy significantly decreases when workers are in complex environments, areas with obstructed views, low lighting conditions, or when the imaging equipment is far from the worker. To address these blind spots in worker monitoring methods using CV, this study aimed to assess the worker’s status using sensors.

To evaluate a worker’s status, accelerometers, gyroscopes, and barometric pressure sensors are primarily used, all of which belong to the category of MEMS sensors. An IMU sensor is a device that integrates these MEMS sensors into a single module to measure changes in the position and posture of an object (or the human body). The IMU sensor includes a three-axis accelerometer and a three-axis gyroscope and can incorporate additional sensors such as a magnetometer or barometric pressure sensor when necessary to obtain diverse motion information. Accordingly, various studies have been conducted using MEMS and IMU sensors to evaluate users’ conditions. For instance, R. Zhong et al. explored gait patterns between young adults and the elderly by having subjects wear a smart bracelet embedded with an accelerometer and gyroscope to record acceleration and Euler angles in real time [3]. S. Chen et al. proposed a method for real-time gait detection and pose estimation when walking on flat terrain and slopes using a single wearable IMU sensor [4]. Additionally, N. Yodpijit et al. designed a system to analyze gait characteristics using smartphone accelerometers as wireless motion sensors. The study quantifies human motion through four main stages: data acquisition, feature extraction, classifier design, and decision-making. Using a peak detection algorithm, the system extracted features such as stride time, stance time, swing time, and cadence to evaluate gait patterns [5].

Studies have also been conducted to monitor poses and behaviors. M. Awais et al. extracted features for sitting, standing, walking, and lying down by having 20 elderly subjects wear accelerometers, gyroscopes, and magnetometers on the chest, wrist, waist, and thigh, and classified these behaviors [6]. H. Li et al. conducted a study to classify actions such as walking, sitting, and bending by having subjects wear IMU sensors composed of three MEMS sensors (accelerometer, gyroscope, magnetometer) on the wrist, waist, and ankle [7]. Y. Lee et al. conducted studies on data poses by measuring inertial data by attaching MEMS sensors to the hands, pelvis, head, and other body parts [8]. N.G. Nia et al. aimed to effectively classify a wide range of human activities using machine learning algorithms, artificial neural networks (ANN), decision tree classifiers (DTC), and k-nearest neighbors (KNN) with IMU sensors composed of three MEMS sensors [9].

In terms of detecting and preventing falls using MEMS sensors, A. Singh et al. reviewed various fall detection systems, classifying them into three main categories: wearable, ambiance-based, and hybrid-sensing detectors. The study analyzed competing sensor technologies, including accelerometers, pressure sensors, radar, and camera-based solutions, and highlighted their strengths and limitations in feature extraction, classification, and real-world applicability [10]. Additionally, N. Shibuya et al. developed a real-time fall detection system using a wearable gait analysis sensor (WGAS) equipped with a tri-axial accelerometer, gyroscopes, and an MSP430 microcontroller. The system utilized a support vector machine (SVM) classifier to extract six features for fall classification, achieving accuracies of 98.8% and 98.7% at different sensor positions and an overall sensitivity of 97.0% [11]. Furthermore, V. B. Semwal et al. placed smartphone MEMS sensors on the abdomen to collect data and applied it to deep learning models to classify the data into fall or non-fall events [12]. H. Choo et al. and S. Hong et al. conducted studies on fall detection and safe behavior monitoring by acquiring data after having workers wear MEMS sensors on safety hooks and equipment to assess the status of workers engaged in scaffolding and high-altitude work [13,14].

Research on developing smart personal protective equipment (PPE) for safety monitoring at construction sites is also actively underway. A. Rashidi et al. conducted a study in which they developed PPE by integrating monitoring systems into gloves and vests, allowing managers to oversee workers [15]. Meanwhile, A. Ojha et al. aimed to measure the overall health of construction workers through wearable biosensors by monitoring three types of physiological signals: PPG, EDA, and ST [16].

However, most of these studies targeted the general public, whose movements often differ from those of construction workers. For example, the general public typically engages in activities close to the ground, such as walking and tripping, whereas construction workers often operate at significant heights using ladders and construction equipment. In addition, when attempting to comprehensively monitor a worker’s health and work status, smartphones or commercial IMU sensors can be bulky, expensive, or uncomfortable to wear, causing inconvenience for workers and disrupting their tasks [17]. Moreover, for greater accuracy, commercial sensors often rely on a single connectivity approach at high sampling rates, making integrated management difficult when multiple sensors are worn on different body parts. In particular, limited server connectivity and transmission protocols present significant challenges to implementing a CPS in large-scale construction sites.

Therefore, this study developed an IMU sensor that includes an accelerometer, gyroscope, barometric pressure, PPG, body temperature, and acoustic sensors. By wearing these sensors on six parts of the worker’s body (head, body, both hands, and both legs), we developed algorithms capable of classifying not only daily movements such as walking, tripping, jumping, and sitting but also specialized tasks like ladder climbing and high-altitude work. Furthermore, by acquiring health data such as heart rate, oxygen saturation, and body temperature, we enabled the assessment of health conditions like heatstroke and cardiac arrest.

## 2. Materials and Methods

### 2.1. Development of Sensors with Enhanced Field Applicability

After analyzing worker behavior patterns and workplace environments, we confirmed that incidents such as falls, slips, falls from heights, collisions, and suffocation due to fires occur frequently. We determined that it is necessary to use sensors specifically capable of monitoring these incidents. Therefore, we selected the following six types of sensors for worker safety monitoring in this study. The definitions of these sensors and their respective monitoring items are as follows:

- Accelerometer: by measuring acceleration, the sensor can assess information such as an object’s tilt (inclination angle) and vibrations. Sudden changes in acceleration values are considered abnormal signals, used to monitor for events like worker falls.
- Gyroscope: this sensor measures the rotational angle per unit time, allowing for detection of changes in orientation and movement.
- PPG sensor: by measuring a worker’s heart rate and oxygen saturation, this sensor aids in identifying critical health risk factors.
- Body temperature sensor: by continuously measuring the worker’s body temperature in real time, this sensor detects sudden changes that may indicate illnesses such as heatstroke or hypothermia.
- Barometric pressure sensor: this sensor measures atmospheric pressure, which helps determine the conditions at the worker’s job site and identify associated risk factors.
- Acoustic sensor: by detecting changes in sound levels during specialized tasks (e.g., welding, cutting), this sensor monitors the worker’s activity duration.

**Figure 1:**
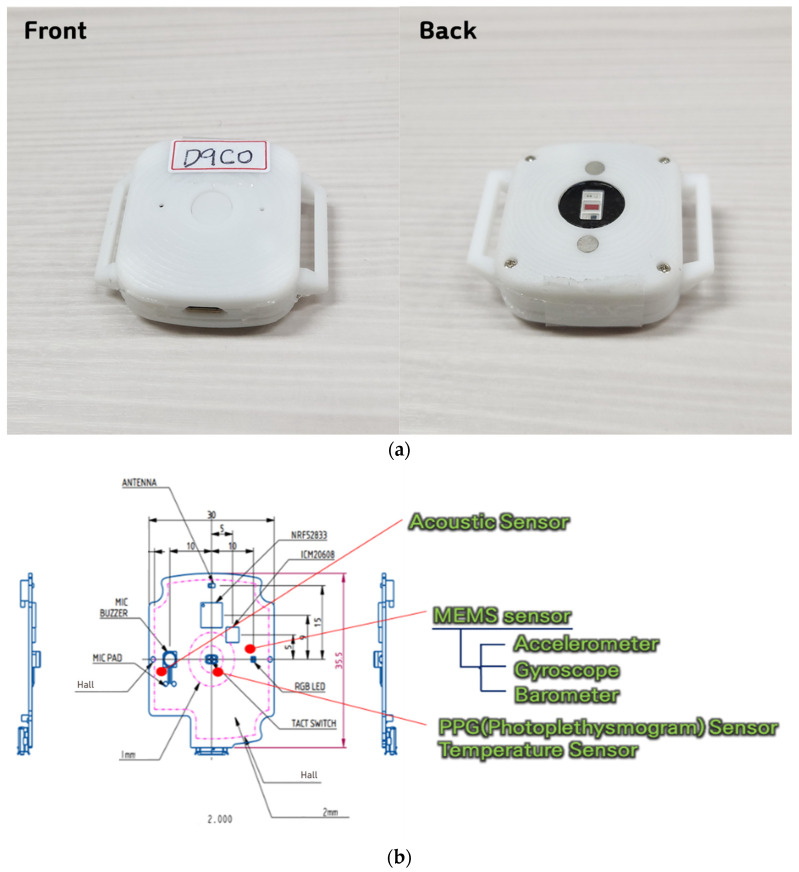
Fabrication of the prototype IMU sensor: (**a**) appearance of the fabricated IMU sensor; (**b**) diagram of the IMU sensor and the positions of the included sensors.

As shown in Figure 1, these six types of sensors were developed and manufactured at a size of 35 × 40 mm to minimize any discomfort workers might experience while wearing them. The prototype IMU sensors were specifically designed to be attachable to a worker’s personal protective equipment.

Each sensor communicates with the main MCU using wired I2C communication. In terms of measurement ranges, the accelerometer operates within ±8 g, the gyroscope within ±1000°/s, and the barometric pressure sensor from 300 to 1200 hPa. The heart rate sensor automatically adjusts its LED current to calibrate for external environmental conditions and skin contact. All sensors are configured to receive data at a rate of 15 times per second, while the heart rate sensor takes measurements after a one-minute interval.

The sensor mainboard is based on a 64 MHz Arm Cortex-M4 and utilizes the IEEE 802.15.4 radio protocol (Bluetooth 5.3). It is powered by a 3.7 V, 500 mAh lithium polymer battery, allowing for approximately 10 h of operation with a charging time of about 3 to 4 h.

### 2.2. Worker Behavior Analysis and Algorithm Development

The IMU sensor used in this study operates at a data collection frequency of 15 times per second, which is relatively low compared to commercial IMU sensors. This limitation makes it challenging to directly apply existing algorithms. Consequently, experiments were conducted that involved data quantification to remove noise, threshold adjustments, and the combination of suitable algorithms. These steps were designed to accurately determine worker status even at low frequencies. In addition, because each worker’s body size, stride length, and work patterns vary depending on the individual and the nature of the work, worker types were categorized to configure the algorithms. Highly compatible algorithms were then developed for application to all workers.

**Figure 2:**
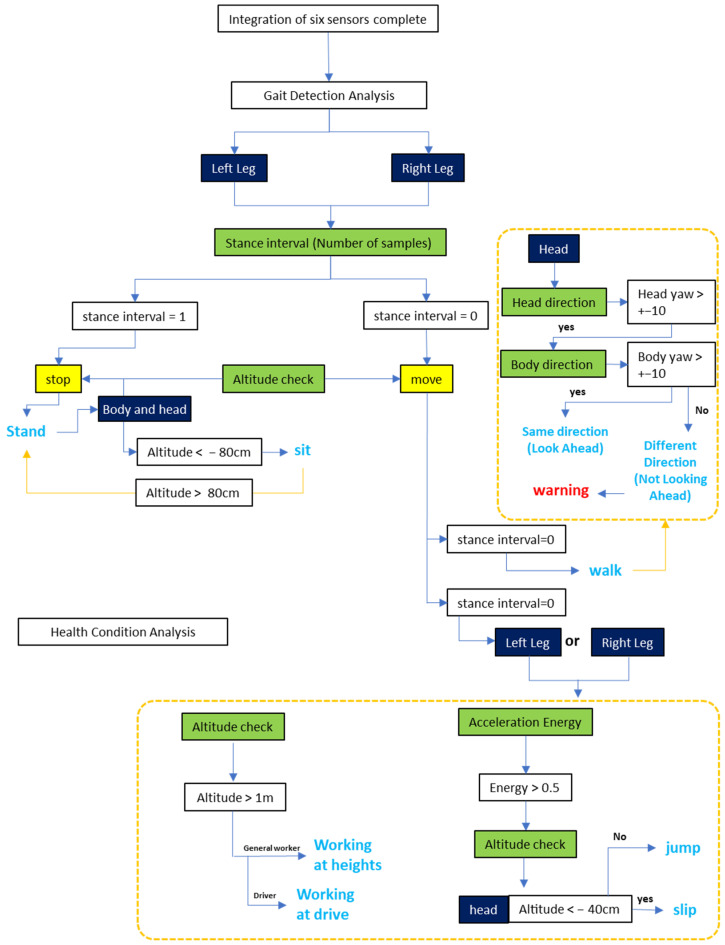
Algorithm flowchart for worker status assessment.

For the experiment, the developed prototype IMU sensor was mounted on the head, body, hands, and legs of five workers. Data were collected by repeating actions such as walking, jumping, standing, sitting, working at height, and looking away from the forward direction, each performed 10 times. Based on the sensing data obtained from each mounting location, optimal filters and data processing methods were applied. The data from each location were then combined according to specific scenarios, and a final algorithm was configured to represent the worker’s status. As shown in the flowchart in Figure 2, this algorithm enables classification across a variety of situations.

Examining the flowchart, data are first collected from sensors attached to the left and right legs to calculate the stance interval. The stance interval indicates whether the worker is stationary or moving at a given location, based on the number of samples recorded during stationary and moving periods. This information provides a basic assessment of whether the worker is standing, moving, or engaging in other activities.

After calculating the stance interval, if minimal movement is detected in both legs, the state is classified as “Standing”. If the worker’s body and head height drop below a certain threshold while in the “Standing” state, the state is classified as “Sitting”. In this study, the threshold was set at 80 cm. If the stance interval and leg movement are active, the state is classified as “Moving”.

In the “Moving” state, the flowchart evaluates whether the worker is looking forward by comparing the directions of the head and body. If these directions are aligned, the state is classified as “Looking Forward”. If they do not align, it is recognized as “Not Looking Forward,” and a warning message is issued.

Additionally, while in the “Moving” state, activities such as “High-Altitude Work,” “Jumping,” and “Falling” are classified by measuring both legs’ altitude and acceleration energy and comparing them against predefined altitude levels and energy thresholds. The algorithms applied to each body part for classification are as Algorithms 1–4.

#### 2.2.1. Forward Attention Algorithm

To process the gyroscope data in the Yaw direction for the head and body areas, the NMNI (noise-matched nonlinear inhibition) filter was applied. The NMNI filter removes noise from a specific gyroscope axis to set a threshold for angular velocity, allowing only signals that exceed this threshold [18]. It operates based on an initial window size (window_size = 50) and angular rate sensitivity (ars = 0.13), using the maximum absolute value of the gyroscope axis data within the given window as the threshold for noise removal.

A Kalman filter was subsequently applied to the data produced by the NMNI filter in order to estimate the state (posture angles) of the head and body. In this study, the Roll, Pitch, and Yaw angles were estimated by integrating accelerometer and gyroscope data. The filter’s state vector is given by [roll, pitch, yaw, bias_x, bias_y, bias_z], where Roll and Pitch are directly calculated from accelerometer data, and Yaw is derived through the integration of gyroscope data. The prediction and update steps of the Kalman filter are as follows:

- Prediction step: predict the next state based on the current state vector and the error covariance matrix.
- Update step: correct the state vector using the measured Roll and Pitch values, and reduce errors by utilizing the measurement noise covariance (R).

The noise covariance matrices of the Kalman filter are defined as Q and R, where Q represents noise occurring during the state transition process, and R represents noise occurring during the measurement process.

Peak detection is then employed to identify significant events (rotations) in the Yaw data of the body and head devices, enabling the detection of moments when the user rotates in a specific direction. The find_peaks function is used to detect peaks in the Yaw data, with a set threshold (threshold = 10) and a minimum distance (distance = 45). This configuration extracts intervals where changes exceeding a certain angle are detected.

**Figure 3:**
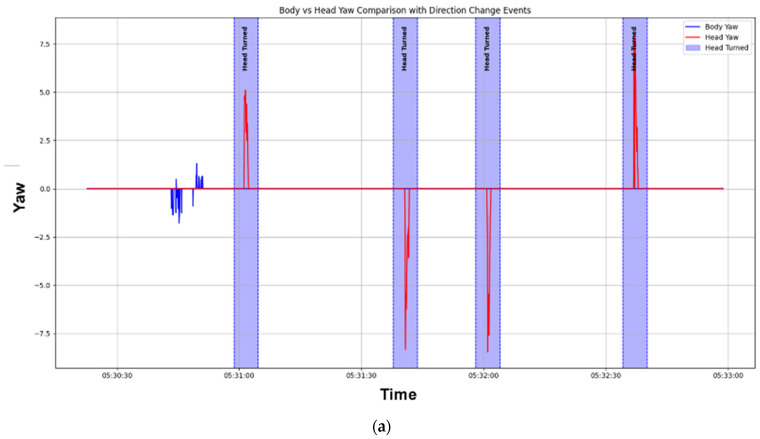

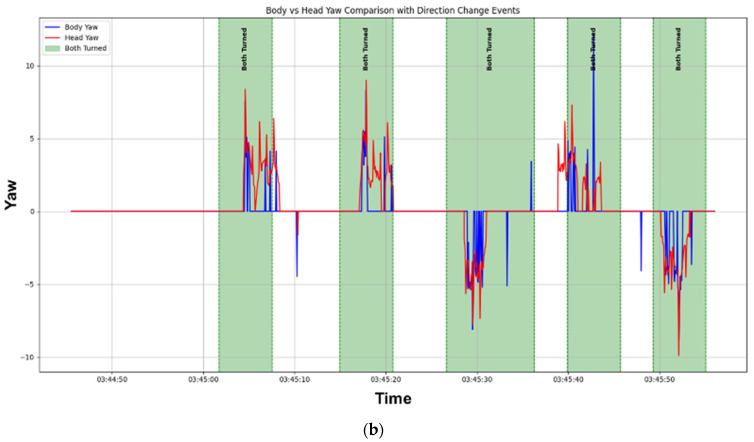
Results of applying the forward gaze algorithm: (**a**) when only the head turns; (**b**) when both body and head turn together (during directional change).

Finally, direction alignment events between the body and head are classified based on the peaks extracted from the Yaw data. As shown in Figure 3, when the worker rotates while walking, and the peak intervals of both devices overlap, the event is classified as “Both Turned”. If the worker rotates only the head while walking, and the peak intervals do not overlap, the event is classified as “Head Turned”.
**Algorithm 1.** Detect forward attention events.Input: body_yaw, head_yaw, body_acc, head_acc, timestamps Output: Forward attention events with timestamps 1. Set parameters threshold = 10  # Yaw angle threshold for peak detection distance = 45   # Minimum sample distance between peaks window_size = 150   # NMNI filter window size ars = 0.13   # Angular rate sensitivity for NMNI filter 2. Apply NMNI filter to Yaw data body_yaw_filtered = apply_nmni(body_yaw, window_size, ars) head_yaw_filtered = apply_nmni(head_yaw, window_size, ars) 3. Initialize Kalman filter for orientation estimation kf = KalmanFilter(Q_acc=0.001, Q_bias=0.03, R=0.01)
4. Estimate orientation with Kalman filterfor each timestamp t: roll, pitch, yaw = estimate_orientation( kf, body_acc[t], head_acc[t], body_yaw_filtered[t], head_yaw_filtered[t], dt) 5. Detect peaks in filtered Yaw data body_peaks = find_peaks(body_yaw_filtered, height=threshold, distance=distance) head_peaks = find_peaks(head_yaw_filtered, height=threshold, distance=distance) 6. Classify events: for each peak in body_peaks: if overlap with head_peaks: classify as Both Turned else: classify as Head Turned 7. Merge overlapping Both Turned events: for each consecutive Both Turned event: if overlap: merge events 8. Output results: for each event: record start_time, end_time from timestamps store event type (“Both Turned” or “Head Turned”)

#### 2.2.2. Gait Detection Algorithm

In the walking detection algorithm, the Kalman filter was also utilized to remove noise and correct sensor data. The state vector of the Kalman filter used in this algorithm consisted of [acc_x, acc_y, acc_z, gyro_x, gyro_y, gyro_z], defining acceleration and angular velocity as the state vector, thereby enhancing the stability of the sensor data [19].

Walking states were detected by analyzing the standard deviations of the filtered acceleration and angular velocity data. The Signal Vector Magnitude (SVM) of the acceleration was calculated, and a sliding window (window_size = 30) was applied to compute the standard deviation (σₐ) for each segment. For the Y-axis angular velocity data of the gyroscope (filtered_gyro_y), the same window size was applied to calculate the standard deviation (σ_ω_).

Based on these two standard deviations, stance intervals were defined. If, within a specific interval, the acceleration standard deviation is less than 0.1 (σₐ < 0.1), and the angular velocity standard deviation is less than 20 (σ_ω_ < 20), that interval is classified as a stance state, facilitating effective detection of walking patterns.

**Figure 4:**
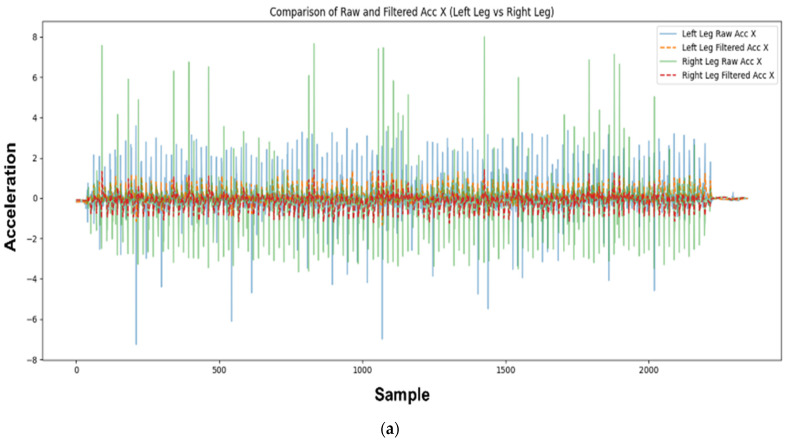

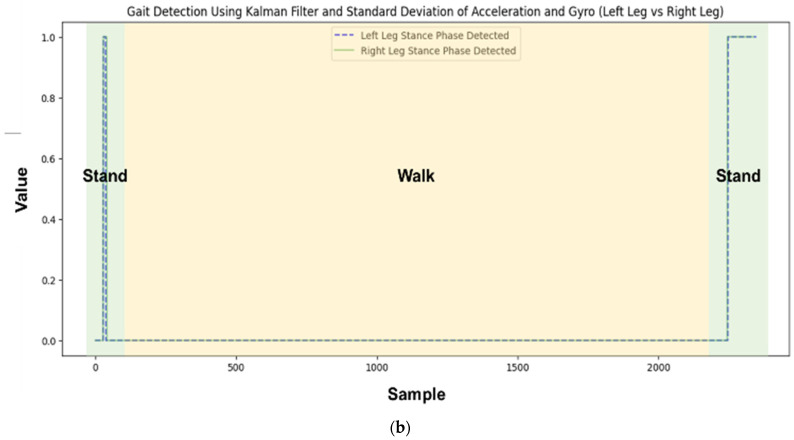
Results of applying the Kalman filter and walking detection: (**a**) result of applying the Kalman filter to raw data; (**b**) walking detection results (1 when there is no foot movement; 0 when there is foot movement).

As shown in Figure 4, after distinguishing between stance and non-stance intervals, the length of each interval was analyzed to understand the periodicity of the walking state. Intervals identified as stance indicate that the movements of both legs have ceased, which corresponds to a “standing” or “sitting” state. When leg movements occur, non-stance intervals appear, allowing the determination of the leg movement state.
**Algorithm 2.** Gait detection using Kalman filter and standard deviation.Input: Accelerometer (acc_x, acc_y, acc_z) and gyroscope (gyro_x, gyro_y, gyro_z) data Output: Stance-phase detection based on filtered acceleration and gyro data 1. Initialize Kalman filter kalman_filter_left = KalmanFilter() kalman_filter_right = KalmanFilter() 2. Filter and calibrate data using Kalman filter for each data point in acc and gyro data: z = [acc_x, acc_y, acc_z, gyro_x, gyro_y, gyro_z]   # Measurement vector kalman_filter.predict()  # Prediction step kalman_filter.update(z)   # Update step save filtered_acc and filtered_gyro   # Store filtered accelerometer and gyroscope data
3. Calculate standard deviation with sliding window acc_magnitude = sqrt(filtered_acc_x^2 + filtered_acc_y^2 + filtered_acc_z^2) sigma_a = rolling_std(acc_magnitude, window=window_size) sigma_omega = rolling_std(filtered_gyro_y, window=window_size) 4. Detect stance phase for each sample: if sigma_a < 0.1 and sigma_omega < 20: classify as Stance Phase else: classify as Non-Stance Phase 5. Analyze stance-phase intervalsfor each classified phase: if Stance Phase: count consecutive samples as Stance Interval else: count consecutive samples as Non-Stance Interval 6. Output results - Print Stance Intervals (number of samples in each stance phase) - Print Non-Stance Intervals (number of samples in each non-stance phase)

#### 2.2.3. Energy Detection Algorithm

During the walking state, an energy detection algorithm was applied to analyze the intensity and periodicity of movements, thereby recognizing worker conditions such as “jumping” and “falling”. To achieve this, a process was designed to calculate energy by removing the DC component based on the root mean square (RMS). Through this algorithm, the magnitude of acceleration changes within a specific time window was measured to detect the worker’s movement patterns [20].

First, to detect worker patterns such as “jumping” and “falling,” the magnitude of the accelerometer sensor data was converted into RMS values. The RMS value represents the average magnitude of the signal, calculated by taking the square root of the sum of the squares of the acceleration vector magnitudes, as defined in Equation (1).

(1) $${RMS}_{acc}=\sqrt{{acc}_{x}^{2}+{acc}_{y}^{2}+{acc}_{z}^{2})}$$

By removing the DC component from the RMS data, the fluctuations in the signal are centralized. As shown in Equation (2), the moving average is subtracted from the RMS values using a sliding window (window_size = 30). This process generates a detrended signal, enabling clearer detection of changes in movement.

(2) $${RMS}_{acc,\;detrended}={RMS}_{acc}-moving\_average({RMS}_{acc})$$

Energy was calculated by quantifying the magnitude changes within a specific window based on the detrended signal. It can be expressed as shown in Equation (3).

(3) $${Energy}_{acc}=\frac{1}{N}\sum_{i=1}^{N}{RMS}_{acc,\;detrended}^{2}$$

**Figure 5:**
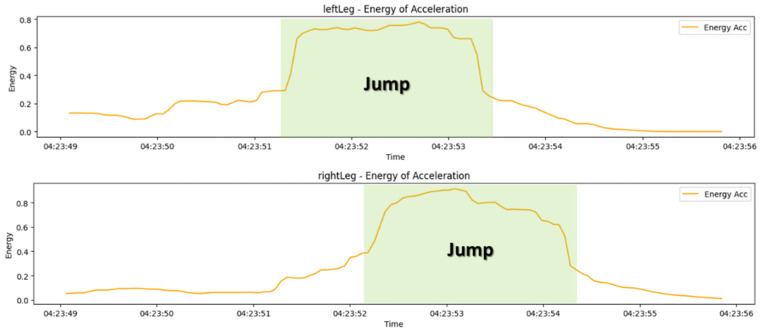
Results of applying the energy detection algorithm during jumping.

Figure 5 presents a graph showing the results of the algorithm applied based on the above equation.
**Algorithm 3.** Accelerometer energy detection algorithm.Input: acc_x, acc_y, acc_z data Output: Energy values for accelerometer data 1. Calculate RMS rms_acc = sqrt(acc_x^2 + acc_y^2 + acc_z^2) 2. Remove DC component rms_acc_detrended = rms_acc–moving_average(rms_acc, window=30) 3. Calculate energy energy_acc = rolling_mean(rms_acc_detrended^2, window=30)

#### 2.2.4. Altitude Detection Algorithm

To estimate the worker’s altitude changes, an extended Kalman filter (EKF) was applied to the barometric sensor data. The EKF, which facilitates state estimation in nonlinear systems, was used to correct errors in the barometric sensor, thereby detecting patterns in the worker’s vertical movement [21].

For altitude estimation, the EKF model’s state vector consists solely of altitude data and is therefore expressed as [altitude]. The initial covariance matrix *p* is assigned large values to account for high uncertainty. The filtering process is as follows. First, the prediction step of the EKF for the state is described by Equations (4) and (5).

(4) $${\hat{x}}_{k|k-1}=A{\hat{x}}_{k-1|k-1}$$

(5) $$P_{k|k-1}=AP_{k-1|k-1}A^{T}+Q$$

In the above equations, *A* is the identity matrix *A* = [1], and *Q* represents the process noise covariance for altitude prediction. Using the altitude data *z* obtained from the barometer, the steps for calculating the Kalman gain and updating the state are presented in Equations (6)–(10).

(6) $$y=z-H{\hat{x}}_{k|k-1}$$

(7) $$S=HP_{k|k-1}H^{T}+R$$

(8) $$K=P_{k|k-1}H^{T}S^{-1}$$

(9) $${\hat{x}}_{k|k}={\hat{x}}_{k|k-1}+Ky$$

(10) $$P_{k|k}=(I-KH)P_{k|k-1}$$

In the above equations, *y* represents the residual, *S* is the covariance, *K* is the Kalman gain, *H* is the measurement matrix, and *R* represents the measurement noise covariance of the barometer.

**Figure 6:**
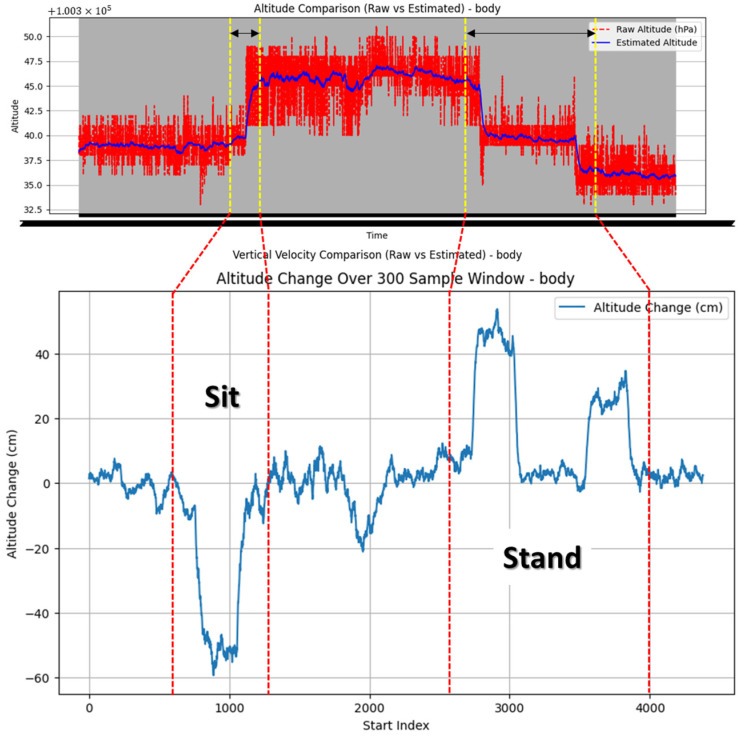
Results of applying the extended Kalman filter to barometric data and estimation of altitude changes.

By applying the EKF to filter the altitude data, we estimated the altitude changes. As shown in Figure 6, the altitude change was calculated as the difference between the starting and ending altitudes within a window of 300 samples.
**Algorithm 4.** EKF for Altitude Estimation.Input: Barometer (hPa) data Output: Altitude estimates over time 1. EKF-based altitude estimation (single state) Initialize altitude state x and covariance *p*
For each barometer data point: - Predict next state: x and *p*
- Update altitude from barometer: z_barometer = [hPa] - Compute Kalman Gain K and update x, *p*
2. Calculate altitude change Convert pressure to altitude (cm) Slide a 300-sample window to compute altitude change: altitude_change = altitude_end–altitude_start

## 3. Results

**Figure 7:**
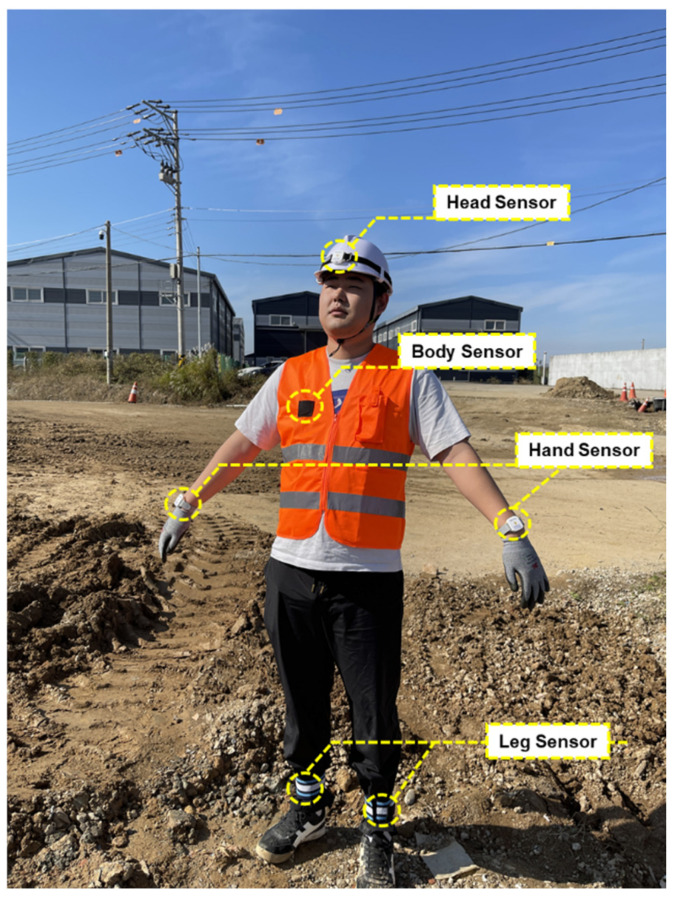
IMU sensor placement by body part.

The developed IMU sensors were attached to the worker’s protective equipment (safety helmet, safety vest, and leg guards), as shown in Figure 7, for experimental purposes. The wrist-mounted IMU sensor was designed in a band form to ensure comfortable use with minimal inconvenience.

**Figure 8:**
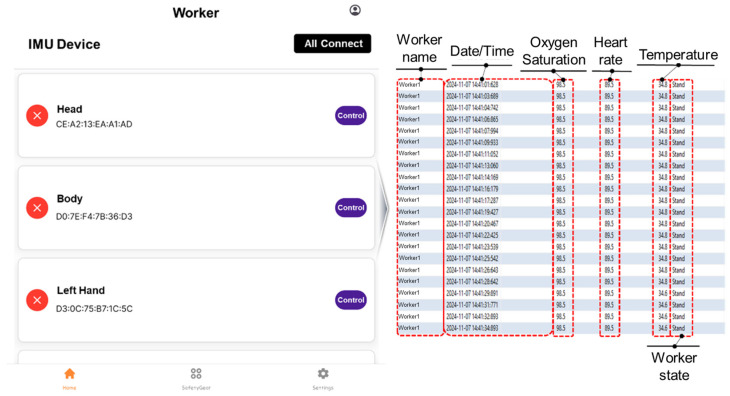
IMU sensor application integration screen and database connection screen.

To integrate the developed IMU sensor data into the CPS, an application was created as shown in Figure 8. The application allows users to input the MAC addresses of the IMU sensors for each body part, enabling Bluetooth connectivity. The sensor data transmitted to the mobile device via Bluetooth are then sent to the server using LTE communication. Additionally, the developed algorithms were implemented within the application to analyze the data collected from specific body parts in real time and determine the worker’s status. The worker’s status, as assessed by the algorithm, is transmitted to a database through the application, where it is used to update the worker’s status in real time within the CPS.

To evaluate the performance of the developed system, experiments were conducted with five workers. Each worker performed every action ten times, producing a total of fifty trials per action. The classification results from these trials are summarized in Table 1.

The sensor data transmitted to the database include information such as oxygen saturation, heart rate, and body temperature, enabling real-time monitoring of workers’ health status. As demonstrated in the experiment, the worker’s condition can also be determined based on the algorithm-assessed worker state (e.g., walking, sitting, jumping, performing high-altitude work, not looking forward, etc.).

In addition, if initial measurements of oxygen saturation, heart rate, or body temperature fall below certain thresholds, the worker’s health status changes from “normal” to “at risk”. This status is displayed on the dashboard, allowing administrators to respond immediately to potential hazards during monitoring.

**Figure 9:**
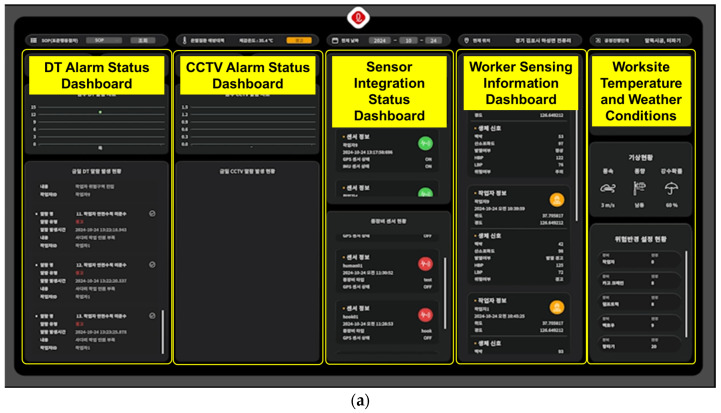

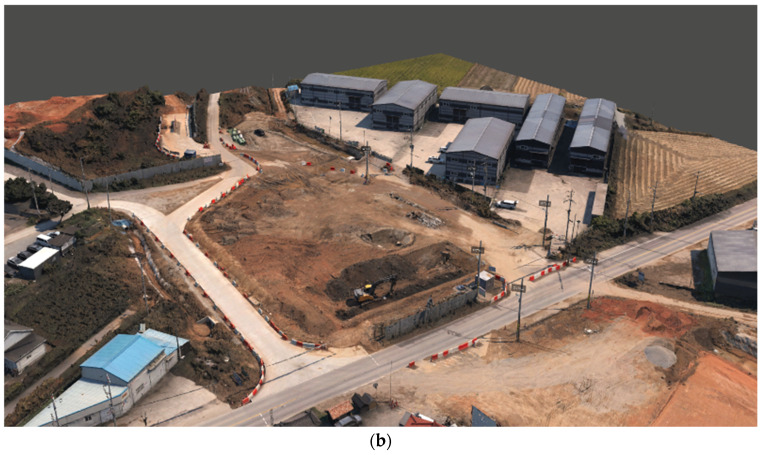
Dashboard and environment setup for CPS implementation: (**a**) CPS dashboard screen layout; (**b**) CPS environment construction scene.

For the data integration experiment, as shown in Figure 9, a dashboard was developed to monitor the worker’s status, and the experimental site was converted into a 3D model using drone imagery. This 3D model was then used to create a CPS environment through the Unity program.

**Figure 10:**
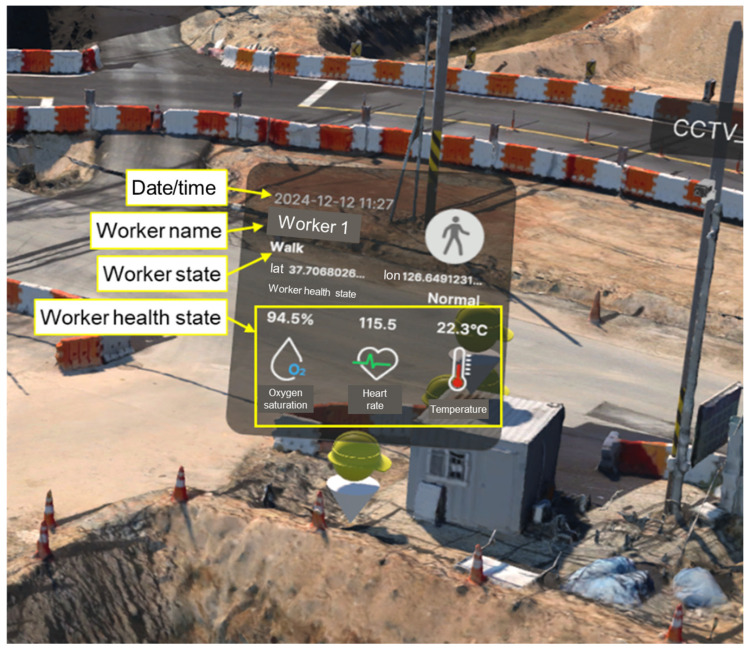
Worker icons and UI in CPS operation.

In the CPS, worker monitoring is displayed through icons, and the user interface (UI) presented to the worker is shown in Figure 10. The UI displays the worker’s name, date and time, status, and health conditions (such as oxygen saturation, heart rate, and body temperature), which match the data transmitted to the database. These data are updated in real time, enabling managers to monitor and identify any abnormal conditions affecting workers.

**Figure 11:**
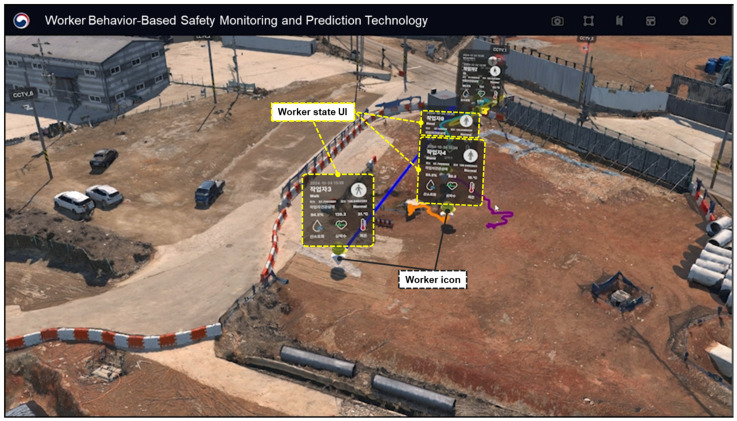
Example of CPS application for multiple workers.

The data shown in Figure 10 are also stored on the dashboard, which is further designed to display various types of information such as sensor connection and operation status, worker status, health information, real-time site temperature, and weather conditions. Figure 11 illustrates a screen where multiple workers are monitored simultaneously. In the event of abnormal situations (e.g., irregular conditions), the dashboard not only stores the relevant information for monitoring but also facilitates proactive on-site actions to prevent accidents. For instance, if abnormal conditions (such as falls or jumps) frequently occur at a specific location, the data generated through this study can be used to either prevent accidents or detect them early through on-site interventions.

## 4. Discussion

This study aimed to develop an IMU sensor for construction and industrial environments and to integrate it into a CPS for monitoring worker behavior under real-world conditions that include the use of protective equipment, uneven terrain, and expansive worksites. During this process, the system achieved an overall accuracy of approximately 90% for recognizing worker actions; however, accuracy for the sitting action was relatively lower. This lower performance may stem from the barometric sensor’s susceptibility to errors caused by uneven ground conditions and fluctuations in temperature and humidity, despite its need for centimeter-level precision. Nevertheless, real-time monitoring of worker behavior through the CPS proved effective in enhancing safety measures and improving operational management efficiency.

Several limitations were identified in this research. First, a delay of approximately 2–3 s occurs between the sensor’s initial detection of movement and its display in the CPS interface. In urgent situations, such as sudden falls, this delay could hinder rapid response. The selected window size for detecting data changes and the latency in transmitting data to the database appear to contribute to this issue. Second, due to the maximum number of Bluetooth devices that can simultaneously connect to an Android system, sensor integration occasionally became unstable. When the IMU sensor’s data transmission rate was increased, the intervals between data arrivals became irregular. Although three IMU sensors transmitted data reliably, connecting six sensors sometimes resulted in two or three failing to meet the set data threshold, thereby reducing the accuracy of the algorithms. A slight delay (on the order of milliseconds) was therefore introduced during transmission to ensure all six IMU sensors could still send quantitative data, establishing an optimal transmission rate of 15 Hz (i.e., 15 data points per second).

Lastly, because sensors may be directly exposed to dust, moisture, and other contaminants in harsh field conditions, those with relatively low IP (ingress protection) ratings risk malfunctioning in such environments. These limitations underscore the need for future research aimed at optimizing algorithms for real-time data processing, refining communication and data transmission methods for multi-sensor integration, and improving sensor casing materials and designs to enhance waterproof and dustproof capabilities, thereby increasing practical applicability in the field.

## 5. Conclusions

This study developed IMU sensors and an application integrated into a CPS to monitor workers’ behaviors in construction and industrial environments. The conclusions of this study are as follows:

1. The IMU sensors used in this research address challenges associated with existing commercial sensors—such as large form factors and difficulties in internal filtering, communication, and server integration—when implementing a CPS.
2. To minimize worker discomfort and enable seamless attachment to personal protective equipment, the sensors were miniaturized and designed for placement on the head, body, hands, and legs. This approach allows for more granular measurement of diverse work activities.
3. The IMU sensors were operated at a relatively low sampling rate of 15 times per second to extend their operating time during work hours, while an algorithm was designed to effectively capture workers’ movements in on-site conditions. In addition, the algorithm retained the flexibility to operate at higher frequencies (e.g., above 50 Hz) for more detailed motion analysis when needed.
4. By attaching sensors to the head, body, both hands, and both legs, worker behaviors—such as walking, jumping, standing, sitting, working at height, and looking away from the forward direction—could be detected with approximately 90% accuracy. The IMU sensors also assessed workers’ health status (e.g., oxygen saturation, heart rate, and temperature) and transmitted these data to a database, which was then linked to the CPS interface, enabling managers to monitor workers in real time.

In conclusion, by developing IMU sensors and implementing an application and CPS equipped with algorithms to recognize worker behaviors, this study facilitates real-time monitoring, and thereby, contributes to enhanced safety management at construction sites.

## Figures and Tables

**Table 1:** Classification results for each action.

Action	Total Trials	Correctly Classified	Accuracy (%)
Walking	50	50	100
Jumping	50	47	94
Standing	50	50	100
High-altitude work	50	45	90
Not looking forward	50	43	86
Sitting	50	40	80

## Data Availability

The algorithm used in this study is included in the manuscript. The sensing data supporting the findings of this study are available from the corresponding author upon reasonable request.
